# Temporomandibular joint dysfunction and orthognathic surgery: a retrospective study

**DOI:** 10.1186/1746-160X-6-27

**Published:** 2010-11-17

**Authors:** Jean-Pascal Dujoncquoy, Joël Ferri, Gwénael Raoul, Johannes Kleinheinz

**Affiliations:** 1 Department of Oral and Maxillo-Facial Surgery at Lille 2 University, France; 2 Department of Cranio-Maxillofacial Surgery University Hospital Muenster, Waldeyerstrasse 30, D-48149 Muenster, Germany

## Abstract

**Background:**

Relations between maxillo-mandibular deformities and TMJ disorders have been the object of different studies in medical literature and there are various opinions concerning the alteration of TMJ dysfunction after orthognathic surgery. The purpose of the present study was to evaluate TMJ disorders changes before and after orthognathic surgery, and to assess the risk of creating new TMJ symptoms on asymptomatic patients.

**Methods:**

A questionnaire was sent to 176 patients operated at the Maxillo-Facial Service of the Lille's 2 Universitary Hospital Center (Chairman Pr Joël Ferri) from 01.01.2006 to 01.01.2008. 57 patients (35 females and 22 males), age range from 16 to 65 years old, filled the questionnaire. The prevalence and the results on pain, sounds, clicking, joint locking, limited mouth opening, and tenseness were evaluated comparing different subgroups of patients.

**Results:**

TMJ symptoms were significantly reduced after treatment for patients with pre-operative symptoms. The overall subjective treatment outcome was: improvement for 80.0% of patients, no change for 16.4% of patients, and an increase of symptoms for 3.6% of them. Thus, most patients were very satisfied with the results. However the appearance of new onset of TMJ symptoms is common. There was no statistical difference in the prevalence of preoperative TMJ symptoms and on postoperative results in class II compared to class III patients.

**Conclusions:**

These observations demonstrate that: there is a high prevalence of TMJ disorders in dysgnathic patients; most of patients with preoperative TMJ signs and symptoms can improve TMJ dysfunction and pain levels can be reduced by orthognathic treatment; a percentage of dysgnathic patients who were preoperatively asymptomatic can develop TMJ disorders after surgery but this risk is low.

## Background

Common symptoms of TMJ (temporo-mandibular joint) disorders are sounds/noises, pain, headaches, limited movement, masticatory difficulty and others. If Surgical-orthodontic treatment is a common and well-accepted treatment approach for patients with maxillo-mandibular discrepancy and aims to produce more harmonious facial skeletal relationships, there is still controversy about the effects of orthognathic surgery on the temporo-mandibular joint and there are few reports on postoperative patient's satisfaction concerning temporo-mandibular symptoms.

Whether for some researchers orthognathic surgical procedures can help in the reduction of TMJ dysfunction [1,2], others investigators have shown that orthognathic surgery in such patients can causes further deleterious effects on the TMJ [3,4].

Furthermore, if for some authors aesthetics and psychosocial factors are the primary motivation for patients who seek orthognathic surgery [5], it is the correction of the functional disability that determines success or failure in this type of treatment, and the TMJs are the foundation for stable results with the orthognathic surgical procedure [4]. The objectives of this retrospective study were to evaluate subjective treatment outcomes in patients with orthognathic surgery, changes in temporo-mandibular joints function and masticatory efficiency, and to evaluate patients' satisfaction. Also it is known that patients' rating of outcome might not correlate with those of clinicians, thus we decided to use a questionnaire to be aware of patients' subjective findings [6].

Many studies showed relief or stability in signs and symptoms of temporomandibular joint [7]. If the patients who are the most satisfied with the treatment outcome are those whose occlusion improves and whose TMD symptoms are relieved most [6], it is known that there is a risk that preoperatively asymptomatic patients can develop TMD symptoms [8]. Therefore, we also sought to assess the appearance of new onset or aggravation of TMJ symptoms after orthognathic surgery.

Besides, TMD prevalence seems to be higher in patients affected by class II and particulary in case of mandibular retrognathism, low angle and deep bite [9,10]; and it was found that treatment outcome concerning TMD is less favorable in patients with mandibular prognathism than with retrognathism [10]. Thus, we tried to compare these two groups of patients.

## Methods

A retrospective study was performed on the osteotomy patients operated on at the Oral and Maxillo-Facial department of the Lille's 2 Universitary Hospital Center in the period from 01.01.06 till 01.01.08. The initial sample consists of 176 consecutive patients identified from the files of our computers and who were treated with a combined orthodontic and surgical approach during this period. We excluded patients with craniofacial anomalies or clefting, those treated by genioplasty only, but not those treated for obstructive sleep apnea syndrome. The orthognathic surgery was only performed for a dentofacial deformity and not only for TMJ internal derangement. A combined surgical and orthodontic approach was performed for each patient and the analysis of Delaire was used to underlining the skeletal deformity and to determine the surgical treatment. The bilateral sagittal split osteotomy technique (BSSO as described by Epker) was used for mandibular displacement, and Le Fort I osteotomy was used to correct the maxillar with no variation in the surgical technique. Only rigid osteosynthesis were used with post-operative intermaxillary fixation for two weeks. All maxillar osteotomies were stabilized using 4 microplates with 1.5 mm diameter screws, and all mandibular osteotomies were stabilized by 4 microplates with 2 mm diameter screws. Cephalometric radiographs were completed a few weeks before surgery and repeated some months after surgery. The addresses of the 181 patients who were operated during the period 2006-2007 were collected and a questionnaire was sent trough post with a letter explaining the importance concerning their perception before and after surgery. Out of the 176 patients, only 57 filled the questionnaire, and the files of these 57 patients were investigated. The questionnaire was designed to assess patient's perceptions and signs and symptoms of TMD before and after surgery. All subjects were informed of the aim of this questionnaire. Included in the questionnaire were closed-form questions related to TMJ symptoms like presence or absence of tmj sounds (clicking, popping or crepitus), pain, tenseness, limited mouth opening, temporomandibular joint locking, deviation on mandibular motion, and also questions related to the use of an orthotic device. Beyond, the overall subjective findings regarding TMJ function was asked. All patients had their surgery completed a minimum of 6 months and maximum of 2.5 years before the time of the survey.

## Results

Of The 176 subjects, 57 returned the questionnaire. The distribution of the patients according to sex and age at the time of the survey was 35 females and 22 males and age range was 16 to 65 years (mean 31.21 years). According to the site of surgery distribution of patients was 9(15.8%) maxilla, 24(42.1%) mandible and 24(42.1%) both.

### The questions and resulting answers are laid out below

#### Did your temporomandibular joints make noises on functioning before and after surgery?

Presurgery: 35 patients answered "none", 16 patients "some", and 6 patients "many". Postsurgery: 26 patients answered "none", 29 patients "some", and 2 patients "many". 15.8% of patients reported improvement, 57.9% no change, and 26.3% an increase. On the 22 patients with sounds pre-surgery, 16 reported TMJ sounds post surgery. On the 35 patients with no sounds pre-surgery, 15 reported new TMJ sounds post surgery.

#### Did you feel pain in the TMJ region before and after surgery?

Presurgery: 4 patients answered "none", 11 patients "some", and 5 patients "many". Postsurgery: 40 patients answered "none", 16 patients "some", and 1 patient "many". 19.3% of patients reported improvement, 63.2% no change, and 17.5% an increase. On the 16 patients with pain pre-surgery, 7 patients reported TMJ pain post-surgery. On the 41 patients with no pain pre-surgery, 10 patients reported new TMJ pain post-surgery.

#### Did you have limited mouth opening (LMO) before and after surgery?

Presurgery: 48 patients answered "none", 6 patients "some", and 3 patients "many". Postsurgery: 42 patients answered "none", 15 patients "some", and 0 patient "many". 14.0% of patients reported improvement, 63.2% no change, and 22.8% an increase. On the 9 patients with LMO pre-surgery, 2 patients reported LMO post-surgery. On the 48 patients with no LMO pre-surgery, 6 patients reported LMO post-surgery.

#### Did you experience temporomandibular joint locking before and after surgery?

Presurgery: 46 patients answered "none", 8 patients "some", and 3 patients "many". Postsurgery: 46 patients answered "none", 10 patients "some", and 1 patient "many". 15.8% of patients reported improvement, 70.2% no change, and 14.0% an increase. On the 11 patients with joint locking pre-surgery, 3 reported joint locking post-surgery. On the 46 patients with no joint locking pre-surgery, 8 reported joint locking post-surgery.

#### Did you feel tenseness when opening the mouth before and after surgery?

Presurgery: 48 patients answered "none", 2 patients "some", and 5 patients "many". Postsurgery: 47 patients answered "none", 7 patients "some", and 1 patient "many". 9.1% of patients reported improvement, 83.6% no change, and 7.3% an increase. On the 7 patients with tenseness pre-surgery, 4 reported TMJ tenseness post-surgery. On the 48 patients with no tenseness pre-surgery, 4 reported TMJ tenseness post-surgery.

#### Did you notice open bite deformity when opening the mouth?

Presurgery: 42 patients answered "no", 5 patients "on the left", and 8 "on the right". Postsurgery: 48 patients answered "no", 6 patients "on the left", and 1 "on the right". 12.3% of patients reported improvement, 85.5% no change, and 1.8% an increase.

#### In case of pre-operative mandibular deviation at mouth opening, can you assess this deviation?

Only 7 patients answered this question with an average deviation of 5 mm.

#### Did you feel clicking when opening or clothing the mouth before and after surgery?

Presurgery: 36 answered "none", 7 "lightly", 5 "some", and 7 patients "many". Postsurgery: 33 answered "none", 12 "lightly", 8 "some", and 2 patient "many". 20.0% of patients reported improvement, 61.8% no change, and 18.2% an increase. On the 19 patients with clicking pre-surgery, 14 reported no TMJ clicking post-surgery. On the 36 patients with no clicking pre-surgery, 9 reported TMJ clicking post-surgery.

#### How do you judge your temporomandibular joint symptoms and feelings, now after surgery compared to prior to surgery?

80.0% of patients reported improvement, 16.4% no change, and 3.6% an increase of symptoms.

#### Did you use an orthotic device (removal plastic appliance) to treat TMJ dysfunction?

Only 8 patients used an orthotic device for 19.3 months on average, 6 of the 8 patients had relief of TMJ symptoms with the orthotic device, and 2 reported no change.

### TMJ Sounds and pain

11 patients (19.3%) had TMJ sounds without TMJ pain pre-surgery, and 5 (8.8%) had TMJ pain without TMJ sounds pre-surgery. 11 (19.3%) patients had both TMJ sounds and pain pre-surgery. 19 patients (28.1%) had TMJ sounds without TMJ pain post-surgery, and 5 (8.8%) had TMJ pain without TMJ sounds post-surgery. 13 (22.8%) patients had both TMJ pain and sounds post-surgery. Thereby these results show a significant increase of TMJ sounds post-surgery, but no significant change on TMJ pain. However, when we consider the 16 patients with pain pre-surgery, 9 (56.3%) of them had a complete relief of pain post-surgery. Also on the 22 patients with sounds pre-surgery, 6 had no sounds post-surgery.

### Distribution of preoperative symptoms

**Figure 1:**
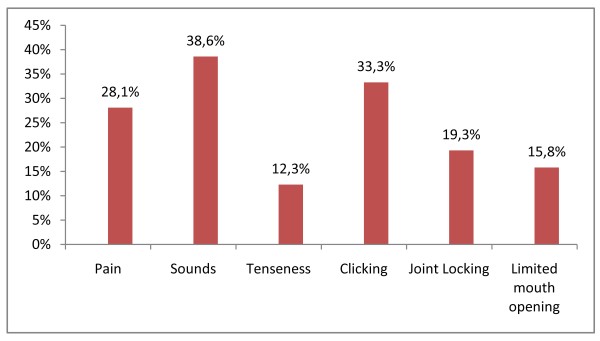
**Distribution of preoperative symptoms**. The figure 1 gives the distribution of preoperative symptoms.

The Figure 1 gives the distribution of preoperative symptoms.

### Preoperatively symptomatic patients who improved TMJ dysfunction postoperatively

**Figure 2:**
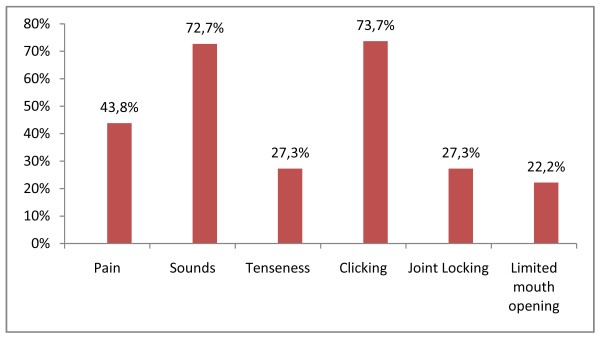
**Preoperatively symptomatic patients who improved TMJ dysfunction postoperatively**. The figure 2 gives the percentage of patient who had a relief of TMJ symptoms.

The Figure 2 gives the percentage of patient who had a relief of TMJ symptoms.

### Preoperatively asymptomatic patients developing new TMJ symptoms postoperatively

**Figure 3:**
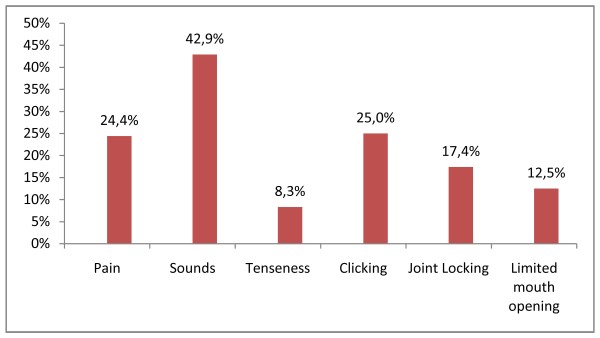
**Preoperatively asymptomatic patients developing new TMJ symptoms postoperatively**. The figure 3 gives the percentage of patient who reported new onset of TMJ symptoms.

The Figure 3 gives the percentage of patient who reported new onset of TMJ symptoms.

### Overall subjective treatment outcome

**Figure 4:**
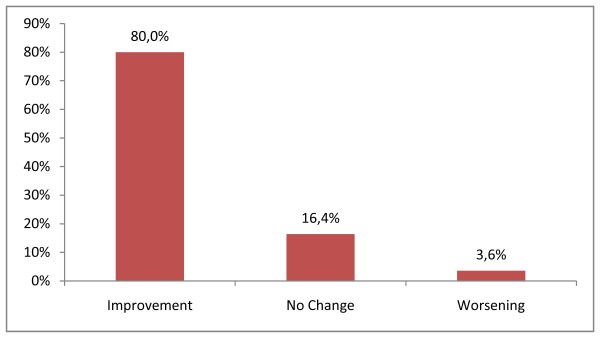
**Overall subjective treatment outcome**. The figure 4 gives the overall subjective treatment outcome on TMJ.

The Figure 4 gives the overall subjective treatment outcome on TMJ.

## Discussion

We investigated the effect of orthognathic surgery on signs and symptoms of TMD after BSSO and/or Le Fort 1 osteotomy.

In our study 56.1% of the 57 patients who returned the questionnaire presented with TMJ symptoms before surgery: 38.6% had sounds, 28.1% had pain, 15.8% had limited mouth opening, 19.3% had temporomandibular joint locking, 12.3% had tenseness when opening the mouth, 22.8% had deviation when opening the mouth, and 33.3% had clicking when opening or clothing the mouth. Whereas only one patient was free of symptoms. This can be assumed to be within a correlation between dysgnathia and TMJ disorders [11]. Our sample of patients has more preoperative symptoms (56.1%) compared to the samples of others studies: Karabouta and Martis reported 40.8% and White and Dolwick reported 49.3% of preoperative TMJ dysfunction, but De Clercq and Abeloos had 26.5% symptoms of dysfunction preoperatively in their sample [1,9,10].

The results of our study show that patients with pre-existing TMJ dysfunction undergoing orthognathic surgery are likely to have significantly improved signs and symptoms of TMJ dysfunction. 16 patients (28.1%) had pain pre-surgery and only 7 of them had pain post-surgery. And for 2 of these 7 patients the frequency and intensity of pain decreased. These results on pain are parallel to some studies but contrasts with the results of others studies: Wolford and al. report 84% patients with TMJ pain after surgery for example [4]. Also, 22 patients had sounds pre-surgery and 16 (72.7%) of them had sounds post-surgery; 9 patients had limited mouth opening pre-surgery and 2 (22.2%) of them had limited mouth opening post-surgery; 11 patients had joint locking pre-surgery and 3 (27.3%) of them had joint locking post surgery: all these results confirm the beneficial effect of orthognathic surgery on patients with TMJ disorders like did others studies [1,12].

On the other hand, some patients may be asymptomatic or have innocuous clinical symptoms. Therefore, we also sought to evaluate the effects of orthognathic surgery on temporomandibular joint in patients with no presurgical TMD: on the 41 patients with no pain pre-surgery, 10 (24.4%) patients reported new TMJ pain; and on the 35 patients with no sounds pre-surgery, 15 (42.9%) reported new TMJ sounds post-surgery. Postoperatively increased loading of the joints occurs until the TMJs soft tissues and muscles reach a state of equilibrium and adapt to the new position, which can explain the onset of TMJ symptoms. We have to inform the patients of this possibility because subjects who believe that they were given too little information tend to be dissatisfied with the treatment results [13]. 48 patients did not have limited mouth opening pre-surgery and only 6 (12.5%) of them had limited mouth opening post-surgery: we can conclude that orthognathic surgery slightly affect the mouth opening. This increase in mandibular hypomobility after orthognathic surgery can be attributed to atrophy and scarring of the muscles and connectives tissues [14]. It is also true concerning joint locking: 46 patients did not have joint locking pre-surgery, and 8 (17.4%) of them had limited joint locking post-surgery. In like manner, our results show that orthognathic surgery can induce some tenseness or clicking on TMJs. The literature agrees with these results: for many authors a percentage of dysgnathic patients who undergo orthognathic surgery develop TMJ disorders after a surgical treatment even if they were asymptomatic [15]. However, some of these new symptoms can be assumed to be within the spontaneous variation: Panula et al. had 3(15%) patients in a control group of 20 patients who developed new symptoms [11]. Furthermore, TMJ disorders are considered a multidimensional condition to which many physical, psychological, and social factors can contribute.

Seven of the nine patients with preoperative limited mouth opening were free of limitation postoperatively, and eight of the eleven patients with joint locking when opening the mouth were completely relieved post-surgery. We can conclude that orthognathic surgery significantly improve mastication and chewing ability which is related to a high satisfaction with the treatment outcome [6].

Some authors propose surgical management of the TMJ pathology as a separate procedure or concomitantly with the orthognathic surgery [4]. Wolford and al. reported that concomitant TMJ (disc repositioning) and orthognathic surgery performed in orthognathic surgery patients with pre-surgical TMJ symptoms resulted in 53% of TMJ pain elimination which is comparable to our results [16]. Therefore, we think that the TMJ surgery may be done as a separate procedure if needed.

For the same author, patients treated within the first 4 years of the onset of TMJ symptoms had better outcomes than did patients who had their TMJ symptoms for longer than 4 years which conduce us to propose earlier surgical treatment.

We used only rigid fixation with post-operative intermaxillary fixation but Buckley et al. have shown no significant difference in the prevalence of TMJ symptoms between patients who have received rigid internal fixation versus nonrigid wire osteosynthesis during BSSO [17]. Also, previous studies showed that the type of fixation in orthognathic surgery does not affect symptoms of TMD [18]. Thus, there is no bias because of our post-operative intermaxillary fixation for two weeks, and our results can be extrapolated to patients with shorter post-operative intermaxillary fixation.

No cases of condylar resorption with posterior shifting of the mandibule were noticed in the present study but it may appear for some authors with a predilection for females [4,19,20]. No cases of fibrous ankylosis were reported but 8 cases were reported by Nitzan and Dolwick [21].

It could be a significant variance on TMJ symptoms changes as a result of the various types of dento-facial deformities corrected, and most of the studies generally showed a greater presence of TMJ disorders in class II patients or mandibular retrognathia [15]. Westermark et al. found more TMJ symptoms in sample of patients with retrognathism than with prognathism, and De Clercq et al. found that TMJ disorders were more prevalent in patient with class II deformities, low angle and deep bite [19,22]. That is supposed to be caused by the high condylar compressive loadings during function and different vector of compressive loading on class II and deep bite patients [8]. But when we divide our patients into different dentofacial deformity subgroup there is no statistical difference in the prevalence of TMJ symptoms preoperativly in class II compared to class III patients. Furthermore, in the subgroup of patients with mandibular retrognathism and low or normal angle, the possibility is high, that TMJ symptoms will improve after surgery with a mandibular advancement [10]. However, our results found a similar improvement of TMJ pain, sounds, tenseness, joint locking, joint clicking, or limited mouth opening in the two subgroups of patients and no more onsets of TMJ symptoms in either of subgroup. Thus, we did not find any connection between TMD and the type of deformity. That result agrees with the studies of Sostmann et al. and Panula et al. [11,23].

Some authors suggested that 92% of orthognathic patients are satisfied with the results [24]. Analysis of the answers of our patients revealed that 80% found the end results satisfying. Therefore, we can conclude that minor problems like temporo-mandibular clicks or discomfort do not appear to affect satisfaction with the outcome. Only 31.5% of the patients responded to the questionnaire. Patients who take care and time to fulfil a questionnaire are more susceptible to be displeased of the outcomes of the surgery. Thus, we can consider that our results about satisfaction are not overvalued, and our results about new postoperative symptoms are not undervalued.

## Conclusion

Patient satisfaction is an important goal in health care, but is difficult to assess and it involves physical and psychological aspects. The results of this study confirm the hypothesis that surgical-orthodontic treatment significantly reduces the prevalence of TMD symptoms. The decrease in TMJ symptoms after surgery can be explained by the improvement in occlusal relationship and the reduction of emotional stress after correction of the jaw deformities. For Onizawa et al. these changes are not due to correction of malocclusion but rather by the effects of the surgery on masticatory muscles [25]. Phakala et al. showed that patients with mainly myogenous origin got more relief than patients with mainly arthrogenous components of TMD [25]. Also, when Harper studied presurgical and postsurgical condylar pathway tracings he found that only 17% of the patients with presurgical TMJ symptoms developed normal condylar pathway tracings after surgery [26].

By these results, our study supports the viewpoint that routine orthognathic surgery can improve TMJ internal derangement with a long-term stability of the orthognathic surgical procedures performed. We can advocate orthognathic surgical procedure for correction of TMJ because it has beneficial effects on TMJ dysfunction. TMD must be closely evaluated, monitored and treated in the orthognathic surgery patient and we have to inform patients of the possibility of new onset of minor TMJ symptoms.

## Competing interests

The authors declare that they have no competing interests.

## Authors' contributions

JF operated 181 included patients in this study. JPD wrote this article. The original idea for this work comes from JF and GR. JK acted as a supervisor. All authors have read and approved the final manuscript.

## References

[B1] Karabouta I Martis C The TMJ dysfunction syndrome before and after sagittal split osteotomy of the rami J Maxillofac Surg 1985 13 185 188 10.1016/S0301-0503(85)80045-X 3860598

[B2] Magnusson T Ahlborg G Svartz K Function of the masticatory system in 20 patients with mandibular hypo- or hyperplasia after correction by a sagittal split osteotomy Int J Oral Maxillofac Surg 1990 19 289 293 10.1016/S0901-5027(05)80423-0 2124601

[B3] Onizawa K Schmelzeisen R Vogt S Alteration of temporomandibular joint symptoms after orthognathic surgery: comparison with healthy volunteers J Oral Maxillofac Surg 1995 53 117 121 discussion 121-123 10.1016/0278-2391(95)90383-6 7830176

[B4] Wolford LM Reiche-Fischel O Mehra P Changes in temporomandibular joint dysfunction after orthognathic surgery J Oral Maxillofac Surg 2003 61 655 660 discussion 661 10.1053/joms.2003.50131 12796870

[B5] Narayanan V Guhan S Sreekumar K Ramadorai A Self-assessment of facial form oral function and psychosocial function before and after orthognathic surgery: a retrospective study Indian J Dent Res 2008 19 12 16 10.4103/0970-9290.38925 18245917

[B6] Pahkala RH Kellokoski JK Surgical-orthodontic treatment and patients' functional and psychosocial well-being Am J Orthod Dentofacial Orthop 2007 132 158 164 10.1016/j.ajodo.2005.09.033 17693364

[B7] Kerstens HC Tuinzing DB Golding RP Van der Kwast WA Inclination of the temporomandibular joint eminence and anterior disc displacement Int J Oral Maxillofac Surg 1989 18 228 232 10.1016/S0901-5027(89)80059-1 2507674

[B8] O'Ryan F Epker BN Surgical orthodontics and the temporomandibular joint. II. Mandibular advancement via modified sagittal split ramus osteotomies Am J Orthod 1983 83 418 427 10.1016/0002-9416(83)90325-1 6573849

[B9] White CS Dolwick MF Prevalence and variance of temporomandibular dysfunction in orthognathic surgery patients Int J Adult Orthodon Orthognath Surg 1992 7 7 14 1453040

[B10] De Clercq CA Abeloos JS Mommaerts MY Neyt LF Temporomandibular joint symptoms in an orthognathic surgery population J Craniomaxillofac Surg 1995 23 195 199 7673448 10.1016/s1010-5182(05)80010-1

[B11] Panula K Somppi M Finne K Oikarinen K Effects of orthognathic surgery on temporomandibular joint dysfunction. A controlled prospective 4-year follow-up study Int J Oral Maxillofac Surg 2000 29 183 187 10.1016/S0901-5027(00)80089-2 10970079

[B12] Kerstens HC Tuinzing DB van der Kwast WA Temporomandibular joint symptoms in orthognathic surgery J Craniomaxillofac Surg 1989 17 215 218 2788177 10.1016/s1010-5182(89)80071-x

[B13] Finlay PM Atkinson JM Moos KF Orthognathic surgery: patient expectations; psychological profile and satisfaction with outcome Br J Oral Maxillofac Surg 1995 33 9 14 10.1016/0266-4356(95)90078-0 7718535

[B14] Storum KA Bell WH Hypomobility after maxillary and mandibular osteotomies Oral Surg Oral Med Oral Pathol 1984 57 7 12 10.1016/0030-4220(84)90249-4 6582438

[B15] Cascone P Di Paolo C Leonardi R Pedullà E Temporomandibular disorders and orthognathic surgery J Craniofac Surg 2008 19 687 692 10.1097/SCS.0b013e3180c31962 18520384

[B16] Wolford LM Karras S Mehra P Concomitant temporomandibular joint and orthognathic surgery: a preliminary report J Oral Maxillofac Surg 2002 60 356 62 discussion 362-363 10.1053/joms.2002.31220 11928087

[B17] Buckley MJ Tulloch JF White RP Jr Tucker MR Complications of orthognathic surgery: a comparison between wire fixation and rigid internal fixation Int J Adult Orthodon Orthognath Surg 1989 4 69 74 2639919

[B18] Nemeth DZ Rodrigues-Garcia RC Sakai S Hatch JP Van Sickels JE Bays RA Clark GM Rugh JD Bilateral sagittal split osteotomy and temporomandibular disorder Oral Surg Oral Med Oral Pathol Oral Radiol Endod 2000 89 29 34 10.1016/S1079-2104(00)80010-4 10630938

[B19] De Clercq CA Neyt LF Mommaerts MY Abeloos JV De Mot BM Condylar resorption in orthognathic surgery: a retrospective study Int J Adult Orthodon Orthognath Surg 1994 9 233 240 7814928

[B20] Crawford JG Stoelinga PJ Blijdorp PA Brouns JJ Stability after reoperation for progressive condylar resorption after orthognathic surgery: report of seven cases J Oral Maxillofac Surg 1994 52 460 466 10.1016/0278-2391(94)90341-7 8169707

[B21] Nitzan DW Dolwick MF Temporomandibular joint fibrous ankylosis following orthognathic surgery: report of eight cases Int J Adult Orthodon Orthognath Surg 1989 4 7 11 2600492

[B22] Westermark A Shayeghi F Thor A Temporomandibular dysfunction in 1,516 patients before and after orthognathic surgery Int J Adult Orthodon Orthognath Surg 2001 16 145 151 11482293

[B23] Sostmann M Meyer J Berten JL TMJ function following orthognathic surgery Dtsch Stomatol 1991 41 487 489 1818634

[B24] Flanary CM Barnwell GM Jr Alexander JM Patient perceptions of orthognathic surgery Am J Orthod 1985 88 137 145 10.1016/0002-9416(85)90238-6 3861099

[B25] Pahkala R Heino J Effects of sagittal split ramus osteotomy on temporomandibular disorders in seventy-two patients Acta Odontol Scand 2004 62 238 244 10.1080/00016350410001667 15513421

[B26] Harper RP Analysis of temporomandibular joint function after orthognathic surgery using condylar path tracings Am J Orthod Dentofacial Orthop 1990 97 480 488 10.1016/S0889-5406(05)80028-9 2353677

